# Contralateral Noise Stimulation Delays P300 Latency in School-Aged Children

**DOI:** 10.1371/journal.pone.0148360

**Published:** 2016-02-05

**Authors:** Thalita Ubiali, Milaine Dominici Sanfins, Leticia Reis Borges, Maria Francisca Colella-Santos

**Affiliations:** Faculty of Medical Sciences, State University of Campinas, Campinas, São Paulo, Brazil; University of Salamanca- Institute for Neuroscience of Castille and Leon and Medical School, SPAIN

## Abstract

**Background and Objective:**

The auditory cortex modulates auditory afferents through the olivocochlear system, which innervates the outer hair cells and the afferent neurons under the inner hair cells in the cochlea. Most of the studies that investigated the efferent activity in humans focused on evaluating the suppression of the otoacoustic emissions by stimulating the contralateral ear with noise, which assesses the activation of the medial olivocochlear bundle. The neurophysiology and the mechanisms involving efferent activity on higher regions of the auditory pathway, however, are still unknown. Also, the lack of studies investigating the effects of noise on human auditory cortex, especially in peadiatric population, points to the need for recording the late auditory potentials in noise conditions. Assessing the auditory efferents in schoolaged children is highly important due to some of its attributed functions such as selective attention and signal detection in noise, which are important abilities related to the development of language and academic skills. For this reason, the aim of the present study was to evaluate the effects of noise on P300 responses of children with normal hearing.

**Methods:**

P300 was recorded in 27 children aged from 8 to 14 years with normal hearing in two conditions: with and whitout contralateral white noise stimulation.

**Results:**

P300 latencies were significantly longer at the presence of contralateral noise. No significant changes were observed for the amplitude values.

**Conclusion:**

Contralateral white noise stimulation delayed P300 latency in a group of school-aged children with normal hearing. These results suggest a possible influence of the medial olivocochlear activation on P300 responses under noise condition.

## Introduction

The central auditory system is not only composed by ascending pathways, but also by an extensive corticofugal system projecting from the cortex towards the cochlea [1, 2]. Previous neuroanatomical, histological and physiological studies performed in animals have demonstrated that fibers from the primary, secondary and association areas of the auditory cortex project to the medial geniculate body (MGB), inferior colliculus (IC), superior olivary complex (SOC) and the cochlear nucleus [3, 4, 5, 6, 7, 8, 9] through topographically organized connections [6, 8]. The efferent connection with the cochlea occurs through the olivocochlear system, which has ipsi and contralateral projections to the inner ear. From the SOC, the lateral olivocochlear bundle (LOC) innervates the ipsilateral afferent auditory fibers under the inner hair cells (IHC), while most of the medial olivocochlear neurons (MOC) cross to the contralateral cochlea and terminate on the body of the outer hair cells (OHC) [10,11].

A few functions have been attributed to the efferent system such as: protection against harmful acoustic over-stimulation, modulation of the micromechanicals of the cochlea, selective attention, and signal detection in noise [12, 13, 14, 15, 16, 17, 18, 19, 20]. However, the functional role of the auditory efferent system and its physiology has not been completely estabilished, especially on higher regions of the central auditory neural system (CANS) [8, 21]. The caudal portion of the CANS, though, has been better studied and there is well-documented evidence that the medial olivocochlear system plays an important role on the modulation of sound amplification characteristics of the cochlea [16, 17, 22].

Many researchers have studied the auditory efferent activity by using electrical and acoustic stimulation of the medial olivocochlear bundle, which induces an inhibitory effect in the contralateral cochlea by reducing the OHC electromechanicals [13, 14, 22, 23]. This inhibitory effect is called the “suppression effect” and it has been intensively studied in humans through the recording of the otoacoustic emissions (OAE) when introducing a noise stimulus to the contralateral ear [13, 22, 24, 25, 26]. Noise stimulation activates MOC bundle in a reflexive manner, which some authors have called the “MOC reflex” and which can be observed by a reduction of OAE responses [13, 18].

The functional consequences of MOC activation are still debated [27, 28, 29]. Many authors have described contradictory results, especially when attempting to correlate the suppression of the OAE with speech-in-noise perceptual measurements [28, 30, 31, 32]. Besides some methodological issues (related to the OAEs collecting parameters) that have been pointed out in the literature [29, 32], de Boer et al. [28] mentions that the conflicting data might also be due to a MOC system’s dependence on attentional and experience-related factors, which suggests that MOC activation on speech-in-noise discrimination may not only be reflexive, but also modulated by a selective and attention-driven mode of operation of the MOC system [28]. Thereby, higher auditory structures in the brain would recruit MOC-mediated mechanisms in specific listening situations [32].

There is some evidence that cortical activity can modulate the auditory sensory function via a cortico-olivocochlear pathway, which regulates cochlear responses and the auditory-nerve afferent firing [33, 34]. Studies using pharmacological cortical deactivation were conducted in animals and both reductions and increases in the cochlear microphonics and the auditory-nerve compound action potentials were observed [34, 35]. Electrical micro-stimulation of chinchillas’s auditory cortex enhanced the suppressive effects of the acoustic evoked olivocochlear reflex [21], and electrical stimulation of the primary and secondary contralateral auditory cortex in epileptic patients reduced OAE amplitude while no significant changes were seen when non-auditory cortical areas were stimulated [27]. All these studies, however, are highly invasive and therefore hardly replicable in humans, especially in clinical settings.

The late auditory evoked potentials provide a non-invasive technique that reflects the neuroelectrical activity in the cortex as a response to a given stimulus [36]. There are very few studies in literature that recorded the late auditory potentials (LAP) and used masking broadband noise stimulation [25, 37, 38, 39, 40, 41], which could provide some insight about the effects of olivocochlear activity on human auditory processing. Salo et al. [39] recorded the LAP in normal hearing subjects and observed N1 amplitude reduction and P2 amplitude increase in the assessment with noise. Schochat et al. [25], on the other hand, found amplitude reductions for waves N1 and P2 when the assessment was repeated applying white noise (WN) to the contralateral ear. Regarding P300, Krishnamurti [41] found increased latencies and reduced amplitudes for P300 in the presence of contralateral noise in a group of adults with auditory processing disorder (APD) but no significant changes were seen in a group of normal hearing adults. In contrast to this data, Polich et al. [37] and Salisbury et al. [38] verified latency increase for P300 wave in the presence of noise in adults with normal hearing. The studies of Schochat et al. [25] and Rabelo et al. [40], however, found no significant changes for P300 values in the assessment with contralateral noise for normal hearing subjects while Rabelo et al. [40] observed that P300 latency increased in the presence of contralateral noise in a group of professional musicians. Nevertheless, the small number of studies and the contradictory results make it still not possible to determine the effects of the olivocochlear activity on cortical areas of the CANS. Besides that, those studies were conducted in adults and as far as we know there is no published research that investigated the effects of contralateral noise stimulation on the LAP in the pediatric population.

The importance of assessing the auditory function in children and adolescents relies on the existence of an association between the learning difficulties and dysfunctions on the processing of acoustic signals along the CANS [42, 43, 44, 45, 46]. Furtheremore, some of the functions attributed to the efferent system are related to selective attention and signal detection in noise [17, 30, 47], which are important auditory abilities for schoolaged children in academic environments. Understanding the functioning of the CANS in the presence of noise, even at higher stages of the auditory pathway, can help on the development of future assessment tools and individualized intervention planning. Also, the late auditory potentials can provide electrophysiological evidence on how noise stimulation affects the processing of acoustic signals in the human auditory cortex, and because the LAP are highly sensitive to electrophysiological changes, they can constitute potential objective measures on the monitoring of a variety of therapeutic processes.

Therefore, in order to investigate the effects of noise on higher structures of the auditory system, we aimed to examine P300 latencies and amplitudes in two different assessment conditions: with and without the presentation of white noise to the contralateral ear. We hypothesized that stimulation by noise could modify the event-related potential P300, which is a cognitive potential that demands discrimination and attention [36]. Thus, the aim of this study was to examine the effects of contralateral noise stimulation on P300 responses of children with normal hearing.

## Methods

### Ethics statement

This study was approved by the Faculty of Medical Sciences Ethics in Research Committee, State University of Campinas (UNICAMP), Campinas, São Paulo, Brazil, under the protocol number 860/2008. Data was collected from October 2013 to November 2014 at the Laboratory of Audiology, Center for Studies and Research on Rehabilitation 'Prof. Dr. Gabriel Porto’ of the Faculty of Medical Sciences, Unicamp (Cepre-FCM/UNICAMP). Written informed consent was obtained from the parents of all children prior to data collection.

### Participants

Twenty-seven children aged from 8 to 14 years old, 6 male and 21 female, were enrolled in the study. All participants were Brazilian-Portuguese native speakers, right handed, typically developing and had normal hearing. Inclusion criteria were defined as: normal hearing as assessed by pure tone threshold audiometry, speech audiometry, tympanometry and contralateral acoustic reflexes [48, 49], normal responses to ABR for waves I, III and V (Biologic NavigatorPro^®^), no history of neurological disorders and no language or learning complaints as informed by children’s parents and teachers. Those children who presented alterations in one or more of the above auditory assessment procedures were not included in the study and were referred to the Otorhinolaryngology’ Service of the University Hospital.

### Procedures and measures

**Fig 1:**
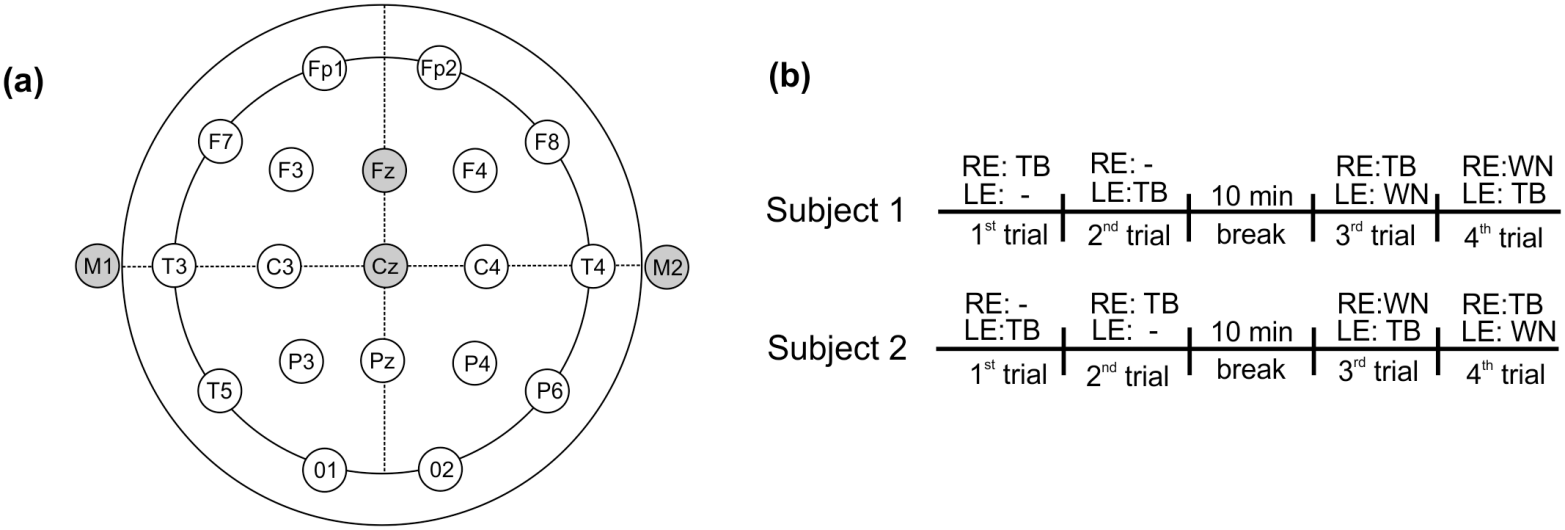
(A) An schematic illustration showing the electrodes setup based on the 10–20 system. (B) Schematic diagram of the time course of the experiments. The order of the tested ear was alternated between subjects, which means that if subject 1 was tested on the right ear (RE) first, the next subject was tested in the opposite order.

P300 was recorded in a sound-attenuated and electrically shielded room in which subjects sat on a comfortable chair. Surface electrodes were placed on the subject’s head with impedance values maintained at below than 3kΩ and inter-electrodes difference under 2KΩ. The active electrode was positioned on the vertex (Cz), the reference electrodes on the right (M2) and left (M1) mastoids and the ground electrode at Fz position (Fig 1A), according to the 10–20 system [50]. The equipment used was a 2-channel electroneuromyograph (Biologic NavigatorPro) and a bandpass filter of 1–30 Hz was used. The elicitor stimulus was delivered monoaurally through insert earphones at 75 dB HL and the oddball paradigm was used to elicit P300. The acoustic stimulus was the tone burst (TB) at the frequency of 2 KHz (100 ms duration) for the infrequent stimulus (target), presented randomly at a probability of 20% and mixed with a frequent tone burst of 1 KHz (non-target), 50 ms duration, presented with 80% probability. Stimulus rate was one stimulus per second, with a total of 300 sweeps. Children were instructed to mentally count the target tone every time they discriminated it, so the examiner was able to guarantee they performed the task properly by asking them how many targets they counted at the end of the evaluation. In addition, children were asked to keep their eyes closed in order to avoid eye movement artifacts. Also, changes in subject’s position (by changing the chair’s recline angles) were tested for those who showed myogenic artifacts, with the purpose of controling adequate recording conditions. After the conventional recording, there was a 10-minute break and then the assessment was repeated with the introduction of contralateral masking noise during all the recording time (Fig 1B). Noise was delivered continuously through insert earphones in the contralateral ear at 75 dB HL and the type of noise used was the white noise (with equal energy distributed to all frequencies from 250 to 6000 Hz) generated by the Biologic Navigator Pro system.

A 700 msec time window was used and analysis was based on the numerical values of the latencies (ms) and amplitudes (μV) in both evaluation conditions: in the presence and in the absence of contralateral noise. P300 was identified as a positive deflection after N1-P2-N2 complex [36, 50]. For these analyses, target wave was subtracted from the nontarget, and latency was set at the most positive peak in the resulting wave.

### Statistical analyses

Kolmogorov-Smirnov test was applied to verify data normal distribution. Student’s t-test for dependent samples and Wilcoxon test for paired samples were used for right and left ears comparison. Mann-Whitney test and Student’s t-test were used for sex comparison and Wilcoxon test for paired samples was used when comparing both assessment conditions: with and without noise. Pearson’s correlations were calculated to determine the relationship between results and age. The level of significance was set at 5% and statistically significant values were marked in bold (p ≤ 0.05). Statistical analyses were performed using SAS 9.2 software.

## Results

Table 1 displays the descriptive statistics of P300 latency and amplitude values with and without contralateral noise stimulation (CN) for the right and left ears. There was no statistically significant difference between ears for any of the studied measures (latency and amplitude) at any of the testing conditions (with and whitout noise). Therefore, the data obtained for each ear was combined on the next analysis [n = 54 ears (female n = 42 ears; male = 12 ears)].

**Table 1:** Comparison between the right and left ears for P300 latency (ms) and amplitude (μV) values in both assessment conditions: with and without contralateral noise (CN) stimulation.

	**Right n = 27**			**Left n = 27**			
	Mean	SD	Median	Mean	SD	Median	*p*-value
Latency (ms) Without CN	320.9	27.1	319.8	325.1	30.1	321.9	0.546
Amplitude (μV) Without CN*	4.4	1.6	3.7	4.9	2.3	3.8	0.9907
Latency (ms) With CN	351.6	46.7	356.3	360.5	36.9	350	0.2733
Amplitude (μV) With CN	5.2	3.2	3.7	5.0	2.2	4.1	0.7525
	**Right n = 27**			**Left n = 27**			
	Mean	SD	Median	Mean	SD	Median	*p*-value
Latency (ms) Without CN	320.9	27.1	319.8	325.1	30.1	321.9	0.546
Amplitude (μV)* Without CN	4.4	1.6	3.7	4.9	2.3	3.8	0.9907
Latency (ms) With CN	351.6	46.7	356.3	360.5	36.9	350	0.2733
Amplitude (μV) With CN	5.2	3.2	3.7	5.0	2.2	4.1	0.7525

Student’s t-test for paired samples

*Wilcoxon test for paired samples

SD = standard deviation

Table 2 shows the descriptive statistics of P300 latencies and amplitudes for the variable sex in both assessment conditions: with and without contralateral noise (CN) stimulation. There were no significant differences between male and female subjects for both latency and amplitude measures in both testing conditions.

**Table 2:** Comparison between female and male results regarding to P300 latency (ms) and amplitude (μV) measures in both assessment conditions: with and without contralateral noise (CN) stimulation.

	Female n = 42 ears			Male n = 12 ears			
	Mean	SD	Median	Mean	SD	Median	*p*-value
Latency (ms) Without CN*	319.65	29.51	320.35	334.79	21.38	337.01	0.1045
Amplitude (μV) Without CN	4.57	1.74	3.74	4.93	2.79	3.73	0.5557
Latency (ms) With CN	349.46	35.85	343.05	378.91	54.22	369.28	0.1061
Amplitude (μV) With CN	4.49	1.63	3.77	7.23	4.41	6.21	0.127

Mann-Whitney test / *Student's t-test / SD = standard deviation

**Fig 2:**
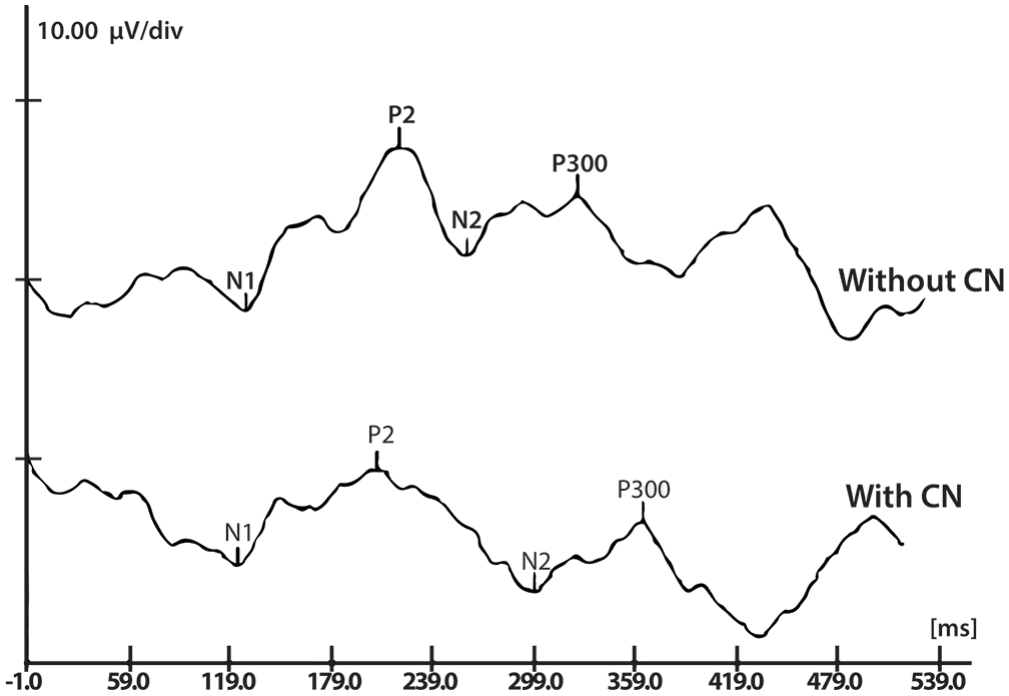
P300 waveforms without and with contralateral noise. CN = contralateral noise; Latency Without CN = 332.98 ms; Amplitude Without CN = 3.00 μV; Latency With CN = 371.88 ms; Amplitude With CN = 3.19 μV.

Table 3 shows a comparison between the assessment conditions (in the presence and in the absence of contralateral noise). Wilcoxon test for paired samples showed significantly greater mean values for P300 lantency (p<0.0001) in the presence of noise. Mean amplitude values were greater in the presence of CN when compared to the mean amplitude values without CN, but this difference was not statistically significant (*p* = 0.566). Fig 2 displays an example of a P300 wave in the absence of noise and an example of a P300 wave in the presence of noise, obtained from one subject.

**Table 3:** Comparison between the conditions with and without noise for P300 latency (ms) and amplitude (μV) values.

	Without CN			With CN			
	Mean	SD	Median	Mean	SD	Median	
**Latency (ms)**	323.01	28.44	321.39	356.00	41.92	353.15	**< 0.0001**
**Amplitude**	4.65	1.99	3.73	5.10	2.72	3.88	0.566

n = 54 ears / Wilcoxon test for paired samples

Table 4 shows the correlations between age and P300 measures [latency (ms) and amplitude (μV)] in the assessment conditions with and without CN. There was a weak positive significant correlation for the variable latency in the testing condition with contralateral noise.

**Table 4:** Pearson's Correlation Coefficient between age and P300 measures [latency (ms) and amplitude (μV)].

	*p-value*	R
Latency Without CN	*0*.*3365*	0.133
Amplitude Without CN	*0*.*3615*	-0.127
Latency With CN	*0*.0474*	0.271*
Amplitude With CN	*0*.*9829*	0.003

*week, positive significant correlation

Table 5 shows the descriptive statistics of the differences between the values derived in the two conditions (with noise *minus* without noise) for the variables latency (ms) and amplitude (μV).

**Table 5:** Descriptive statistics of the difference between the values obtained with and without noise (with noise *minus* without noise) for P300 latency (ms) and amplitude (μV) measures.

(n = 54)	Latency (ms)	Amplitude (μV)
Mean	32.98	0.44
SD	40.19	2.83
Minimum	-70.96	-5.79
Median	23.42	0.17
Maximum	145.74	11.07

SD = Standard Deviation

## Discussion

In this study, P300 responses to target tones were different in the presence and absence of contralateral noise stimulation (Table 3), which is in accordance with previous studies that also reported greater mean latency in the noise condition [37] with no signifficant changes in the amplitude mean [38, 40]. These results suggest that latency measures may show more vulnerability to the effects of noise on P300 responses than the amplitude [38, 40]. Contrary to these findings, however, a study by Schochat et al. [25] reported no changes on P300 latencies in the presence of noise, though the authors observed significant reduction on OAE responses in the same subjects.

### The effects of noise on P300 latency

Table 5 shows the magnitude of the changes (noise *minus* no-noise difference) between with and without noise conditions for latency and amplitude measures. Noise stimulation delayed P300 latency in our sample in 32.98 ms with a standard deviation of 40.19 ms. To our knowledge, there are no studies in the literature reporting the effects of noise on P300 responses in samples of children to which we could compare our results. In the study of Rabelo et al. [40], there was a latency increase of 27.80 ms (SD = 29.31; right ear) and 21.50 ms (SD = 37.50; left ear) in the studied musicians group and an overall decrease of 3.80 ms (SD = 34.82) and 1.00 ms (SD = 30.67) for the right and left ears, respectively, in the non-musicians group. Also, Polich et al. [37] reported a P300 latency delay of about 10 ms in the condition with noise. Other previous studies that recorded P300 in noise conditions [25, 38] did not report the mean of the difference of the results obtained with and without noise. It should be mentioned that several studies about test-retest stability have demonstrated that P300 is a steady potential regarding the latency measure [51, 52, 53, 54] and therefore we could demonstrate that the latency changes observed in the present results are most likely due to the effects of noise than a normal variability between test-retest conditions.

### Acoustic differences in the stimulation protocols among studies

The contradictory results between the study of Schochat et al. [25] and the present research might be related to some differences on P300 collecting parameters. Though acoustic stimuli variations might not be expected to affect P300 at suprathreshold intensities [36], it can still be slightly affected by a few acoustic parameters [38]. Polich et al. [37] found decreased latencies as the frequency of target tone was increased. According to the authors, this might be related to task discrimination difficulty, which means that the greater the difference between target and standard tones the less difficult the discrimination can be. Our study used 2000 Hz as target while Schochat et al. [25] and Rabello et al. [40] used 1500 Hz. Rabello et al. [40], however, found significant latency increase in the noise condition only for the musicians group, not for controls, which could be justified by the greater effectiveness in discriminating the target stimulus by musicians over non-musicians [40]. Polich et al. [37] and Salisbury et al. [38] also used the target tone at 1500 Hz, but contrary to the above-mentioned studies, Polich et al. [37] and Salisbury et al. [38] recorded P300 using binaural stimulation, which can also affect P300 responses [37]. Krishnamurti [41] also used binaural stimulation in the condition without noise (using 4000 Hz as target tone and 3000 Hz as non-target tone), but on the contrary to the present results, Krishnamurti [41] found P300 latency increase only in the group of adults with auditory processing deficits while no changes were seen in the control group (adults with no hearing complaints).

Differences on the noise stimulation levels and consequently on the signal-to-noise ratio (SNR) may have also contributed to the divergent results [39, 55]. Schochat et al. [25] used contralateral white noise delivered at 60 dB HL, which consists in a +10 dBHL signal-to-noise ratio. In the present study, white noise was delivered at 75 dBHL (SNR = 0), while Rabello et al. [40] stimulated the contralateral ear with noise at 70 dB HL (SNR = 0 dB) and Polich et al. [37] also obtained a signal-to-noise ratio of 0 dB with noise and tones delivered binaurally at 60 dBHL. Salisbury et al. [38] used white noise at 70 dB SPL and tones at 97 dB SPL (SNR = +27 dBSPL) binaurally presented. Krishnamurti [41], on the other hand, used monaurally presented tone burst stimuli at 70 dBHL and contralateral white noise at 40 dBSPL. Those noise intensity differences may suggest that higher noise levels or lower SNR might be needed to reveal changes on P300 responses, unlike what seems to occur with the suppression of the OAE. There are studies in the literature reporting that contralateral masking was sufficient to attenuate OAEs even with contralateral intensities as low as 40 dBHL [25, 28, 30, 56]. All those recording parameters differences may have contributed to some of the inconsistent results among studies.

### Medial olivocochlear reflex—the inhibitory effect

One possible explanation for the latency increase observed in the present study may be related to the inhibitory effect caused by the activation of the olivocochlear system [13, 22]. Several researchers studied the inhibitory effect on OAEs by stimulating the contralateral ear with noise [13, 24, 25, 26]. Noise stimulation activates the medial olivocochlear bundle (MOCB) in a reflexive manner, reducing the cochlear amplifier gain and decreasing OAE’s amplitude [13]. Reductions on cochlear micromechanicals may reduce the primary afferent neurons firing [29], which could reflect on longer P300 latencies due to a delay on signal transmission throughout the entire ascending pathway [38, 57].

### Corticofugal influence

The efferent system can also modulate auditory afferents through corticofugal projections [1, 21]. There is anatomical evidence that the auditory descending pathway comprises at least three feedback loops: olivocochlear, colliculo-cochlear, and cortico-olivocochlear [34]. However, though past studies have demonstrated that electrical cortical stimulation and auditory cortex deactivation can modulate coclear responses and the auditory-nerve compound action potential [27, 33, 34], the role and the neurophysiology of cortical activity in noisy listening conditions are still unclear.

Leon et al. [34] suggested that the auditory efferent functions could be classified according to the neural circuit most likely involved. According to the authors, efferent functions comprising brainstem loops would include protection to acoustic trauma and balance of interaural cochlear sensitivity, while functions involving cortico-olivocochlear pathways would be related to control of auditory afferent responses during sleep stages and selective attention. Still, though P300 is an endogenous cognitive potential related to attention [36], it is unkown if higher structures in the efferent paths would be activated by noise, provoking increased P300 latencies as demonstrated in the present study. Nevertheless, our data support previous researches suggesting that the efferent system could be involved (whether by cortico-olivocochlear or by olivocochlear tracts) on the inhibition effect of the event-related potential P300 [37, 38, 40].

### Middle-ear acoustic reflex activation

The middle-ear acoustic reflex (MEMR) is another physiological mechanism that must be taken into consideration when interpreting our data [32]. According to literature, stimulus from 70 dB above an individual’s pure tone thresholds may trigger the MEMR in some subjects [49], which would attenuate sounds audibility by enhancing middle-ear acoustic impedance. In our sample, all subjects had middle-ear acoustic reflexes thresholds for pure-tone stimulus from intensities higher than 80 dBHL and pure-tone audiometry thresholds below 20 dBHL, which suggest that the P300 elicitor stimulus at 75 dBHL could not provoke middle-ear muscle contractions. Nevertheless, literature reports lower MEMR thresholds when they were measured using noise stimulus [58, 59]. As white noise was presented at 75 dBHL in our procedures, there is a high possibility that the MEMR mechanism influenced our results because it was triggered in some of the assessed subjects. Future studies should consider measuring the middle-ear acoustic reflexes using noise stimulus for a cut off exclusion criteria in an attempt to separate MOC and MEMR effects. Separating MOC and MEMR effects, however, may not be such an easy task. Recent research using wideband acoustic immittance procedures have demonstrated extremely low MEMR thresholds than the ones previously reported in the literature by using the current clinical tools [59]. However, according to Keefe, et al. [59], there is a possibility of a contribution from the MOC reflex in those low MEMR thresholds and this possibility raises the question of the extent to which MOC and MEMR effects can be simply separated. Further research is needed to investigate these issues. Nevertheless, the present results did not exclude the possibility of an MEMR contribution and more research should be conducted to understand whether the effects of noise on P300 latencies are due to MOC or MEMR effects, or to some combination of both mechanisms.

### The slower processing hypothesis

An alternative explanation for the changes observed in the P300 latencies in the present work could be related to the difficulties created by the noise condition. The introduction of contralateral noise would make the discrimination task more difficult and therefore result in increased latencies. If this hypothesis is true, delayed P300 latencies could mean that the noise condition can cause slower information and cognitive processing. Also, if this slower processing can occur even in normal developing children (as demonstrated in the current research), the effects of noise in children with learning problems could be even worse. Krishnamurti [41] observed that the noise condition delayed P300 latency (and reduced P300 amplitude) in a group of adults with auditory processing disorder while no significant changes were seen in the studied group of normal hearing adults. According to the author, the competing noise may have resulted in a “line-busy phenomenon” and increased the difficulty in attending and discriminating between target and nontarget tones on the P300 task.

The “slower processing” hypothesis, however, would not explain the delayed P300 latencies demonstrated by the musicians group in the study of Rabelo et al. [40], since musicians are not expected to present worse responses than non-musicians. The literature has shown enhanced electrophysiological responses (in conditions without noise) in musicians when compared to non-musicians [40, 60, 61, 62], suggesting that the reduced latencies observed in musicians may be related to faster stimulus transmission and information processing throughout the CANS, and the higher amplitudes may reflect more neural connections in the auditory pathway [40]. The greater P300 inhibition (delayed latencies) reported by Rabelo et al. [40] in the musicians group may indicate a better signal transmission in the auditory system of musicians not only in the ascending auditory pathway but also in the auditory descending pathway. In fact, the literature reports stronger OAE suppression in musicians [63], supporting the assumption of a more effective signal transmission through the auditory efferent system in musicians.

The different signal-to-noise ratios used by Krishnamurti [41] and Rabelo et al. [40] may have lead to the involvement of different neurophysiological mechanisms. There is a complex relation between olivocochlear recruitment and the activation of corticofugal pathways, and many auditory loops (including afferent and efferent connections) can be involved in the P300 experiments with noise [2].

### The effects of noise on P300 amplitude

Regarding the effects of contralateral noise on P300 amplitude, although mean values were greater in the presence of noise, this effect was not statistically significant in our data (Table 3), which is similar to the findings reported by Salisbury et al. [38]. One possible explanation for the amplitude increase in some of the assessed subjects could be related to the allocation of greater attentional and discrimination resources necessary to respond to targets in the noise condition [38, 57]. The presence of noise may have made the oddball task more difficult so the subjects had to make greater effort to perform the task. According to Salisbury et al. [38], the effects of noise on P300 amplitude could be different if a more complex task was used, such as a three-tone discrimination task, or less readily discriminable target and standard tones. Salisbury and his colleagues [38] also argue that the effects of noise on P300 amplitude appears to be random since twenty-one of forty subjects showed amplitude increase against nineteen that showed amplitude decrease in the presence of noise. This data suggests that subjects may have used different physiological mechanisms to attend to targets in the noise, which may imply different neural substrate than the ones usually activated by noise on the inhibition of the otoacoustic emissions.

### Correlation between latency and age

The present study also showed a positive significant correlation between the variables age and latency in the testing condition with noise (Table 4). Though this correlation was weak, it may indicate a possible maturation of the auditory descending pathways. P300 latencies were longer as age increased, which may be due to a more effective signal transmission through the efferent system, provoking greater inhibition effect in the older subjects. Although we found no correlations on the evaluation without noise, P300 maturation is described in literature showing latency decline with increasing age in children [36, 57]. In the noise condition, we could expect the opposite: latency increasing as age increases as well, if the development of the auditory efferent system would be parallel to the afferent pathway maturation. This hypothesis, however, is mainly speculative and future research should be designed to have it tested.

### Limitations of the study

The present study had a few limitations. Sample size was relatively small for a normative database and the number of girls was much higher than boys due to a recruitment difficulty (only a few boys volunteered for participating in the study and among them, some were excluded due to failling to match the inclusion criteria). Future studies should consider sample size calculation for boys *versus* girls in order to establish a normative database.

### Clinical implications and future researches

Understanding the functioning of the CANS in the presence of noise in normal developing children may allow important insights into how noisy listening situations can impair information processing in school-aged children with auditory processing difficulties. Identifying auditory deficits in children is highly important because impairments on auditory skills have been related to the learning disabilities [42, 43, 44, 45, 46]. Our study presents a first step in developing a new electrophysiological measure for assessing the CANS in school-aged children, especially regarding the auditory efferent processing at higher levels of the system. Future researches should consider a higher number of subjects in order to set normative data to this population. Also, different parameters should be tested, such as the comparison of different noise levels, and the use of other acoustic stimuli, such as speech stimulus. Besides that, our data provides relevant information for a potential clinical applicability of a P300 suppresion protocol in identifying auditory processing deficits in patients who typically perform within normal standards to peripheral and central auditory tests but still have important complaints about hearing in noise [55]. The present results, however, cannot be generalized to adolescents and younger adults due to maturational differences on electrophysiological responses [36, 57]. Future studies should investigate possible age-related effects on the inhibition of the event-related potential P300 in order to set normative database for a larger age range.

## Conclusion

The results obtained in the present study suggest that contralateral white noise stimulation can delay P300 latency in normal hearing children, when assessed by an oddball paradigm with easily discriminable tones. Amplitude measures, however, were not sensitive to reflect the effects of noise on the auditory event-related potential P300.
